# Propranolol (1 mg/kg/day) with intralesional bleomycin versus propranolol monotherapy for infantile hemangioma: a randomized controlled trial

**DOI:** 10.3389/fphar.2025.1710517

**Published:** 2025-11-03

**Authors:** Yanyan Guo, Xinxian Liu, Sicheng He, Bin Zhou

**Affiliations:** Interventional Radiology Department & Hemangioma Department, Wuhan Children’s Hospital (Wuhan Maternity and Child Health Care Hospital), Tongji Medical College, Huazhong University of Science & Technology, Wuhan, Hubei, China

**Keywords:** infantile hemangioma, propranolol, bleomycin, combination therapy, randomized controlled trial, pediatric dermatology

## Abstract

**Objective:**

To evaluate the efficacy and safety of oral propranolol (1 mg/kg/day) combined with intralesional bleomycin injections versus propranolol monotherapy at the same dosage for infantile hemangioma (IH). This study investigates if a low-dose propranolol regimen can be enhanced with local therapy to improve outcomes while maintaining a favorable safety profile.

**Methods:**

This single-center, prospective, randomized controlled trial enrolled 260 infants (aged 3–11 months, mean age 5.34 ± 2.57 months) with IH requiring systemic therapy. Participants were randomly assigned (1:1) to either the combination group (propranolol plus monthly intralesional bleomycin, n = 130) or the monotherapy group (propranolol alone, n = 130). The primary efficacy endpoint was the proportion of patients achieving an excellent therapeutic response (complete regression or marked effectiveness) at 6 months. Secondary outcomes included early therapeutic response, changes in hemangioma color score, tumor volume reduction, Vancouver Scar Scale (VSS) scores, and incidence of adverse events.

**Results:**

Baseline characteristics were comparable. After 6 months, a significantly higher proportion of patients in the combination group achieved the primary endpoint (77.69% vs. 50.00%; P < 0.001). The combination group had higher rates of complete regression (33.07% vs. 15.38%, P = 0.001) and marked effectiveness (44.61% vs. 34.61%, P = 0.083). A superior early response was noted in the combination group, with a more pronounced degree of tumor atrophy within 24 h (P < 0.001). Post-treatment color scores (change from baseline, P < 0.001) and tumor volume (1.63 ± 0.70 cm^3^ vs. 3.27 ± 1.06 cm^3^, P < 0.001) were significantly better in the combination group. VSS scores were significantly lower in the combination group (3.68 ± 0.37 vs. 5.75 ± 0.64; P < 0.001), indicating less scarring. Safety profiles were comparable.

**Conclusion:**

In infants with IH, augmenting a low-dose oral propranolol regimen (1 mg/kg/day) with monthly intralesional bleomycin is significantly more effective than low-dose propranolol monotherapy. This combination strategy accelerates tumor regression and yields superior cosmetic outcomes, all while maintaining a comparable safety profile.

## Introduction

Infantile hemangioma (IH) is the most common benign vascular tumor of infancy, affecting approximately 4%–10% of infants, with a higher prevalence in females, premature infants, and those with low birth weight (Léauté-Labrèze et al., 2017; Sharma et al., 2024). The natural history of IH typically involves a proliferative phase within the first few weeks to months of life, followed by an involution phase that can last several years (Hoeger et al., 2015). While many IHs regress spontaneously, a clinically significant proportion, estimated at 15%–20%, may not resolve completely or can lead to complications such as ulceration, bleeding, functional impairment (e.g., visual or airway obstruction), or significant disfigurement, necessitating active treatment (Lu et al., 2024; Zavras et al., 2020).

Oral propranolol, a non-selective beta-blocker, has revolutionized IH treatment and is now considered the first-line systemic therapy (Krowchuk et al., 2019; Chen et al., 2024). Its mechanisms of action include vasoconstriction, inhibition of angiogenesis (e.g., via downregulation of vascular endothelial growth factor (VEGF) pathways), and induction of endothelial cell apoptosis (Storch and Hoeger, 2010; Lee et al., 2014). While the therapeutic dose of propranolol typically ranges from 1 to 3 mg/kg/day, with 2 mg/kg/day often used for optimal efficacy (Chen et al., 2023), there is a clinical imperative to minimize systemic exposure in young infants to reduce potential adverse effects like hypoglycemia, bradycardia, hypotension, and bronchospasm (Hermans et al., 2024). This has prompted research into the efficacy of lower-dose regimens.

Intralesional bleomycin, an antineoplastic agent with sclerosing properties, has emerged as a therapeutic option for vascular malformations and refractory or complex IHs (Horbach et al., 2016; Kiani et al., 2025). Bleomycin induces endothelial cell damage and promotes local fibrosis, leading to lesion regression (Muir et al., 2004). Local injection offers targeted therapy with higher drug concentrations at the lesion site and potentially fewer systemic side effects compared to systemic administration (Kumar et al., 2021). Previous studies have suggested that combining propranolol with other modalities may enhance efficacy (Guo et al., 2024; Tiwari et al., 2022). However, robust evidence from RCTs evaluating the augmentation of a low-dose propranolol regimen with intralesional bleomycin is still limited.

We hypothesized that a synergistic effect could be achieved; propranolol’s systemic anti-angiogenic action may sensitize the proliferating endothelial cells, making them more susceptible to the potent, localized cytotoxic effects of intralesional bleomycin, thereby accelerating regression and improving final cosmetic outcomes even with a lower systemic propranolol dose. The primary objective of this study was therefore not to challenge the efficacy of standard-dose propranolol, but to determine if a low-dose regimen (1 mg/kg/day) could be significantly augmented by adjuvant intralesional bleomycin to achieve superior efficacy compared to low-dose monotherapy alone.

## Methods

### Study design and oversight

This study was a prospective, randomized, controlled, open-label trial with blinded outcome assessment conducted at Wuhan Children’s Hospital, China. The trial was designed and reported in accordance with the Consolidated Standards of Reporting Trials (CONSORT) 2010 statement (Schulz et al., 2010). The study protocol was approved by the Institutional Ethics Committee of Wuhan Children’s Hospital. All parents or legal guardians provided written informed consent.

### Study participants

Infants with IH were recruited from our hospital between 1 January 2024, and 1 January 2025. All diagnoses were made clinically by two experienced dermatologists. High-frequency ultrasound was used in all cases to confirm the diagnosis, measure tumor dimensions, and rule out other underlying structures, ensuring a homogenous study population.

Inclusion Criteria: (1) age 3–11 months; (2) diagnosed IH requiring treatment (e.g., rapid growth, high-risk location, risk of functional impairment, ulceration, or significant cosmetic concern); (3) no prior treatment for IH; (4) guardians provided informed consent.

Exclusion Criteria: (1) known hypersensitivity to propranolol or bleomycin; (2) congenital or mixed hemangiomas; (3) significant cardiopulmonary, hepatic, or renal dysfunction; (4) other severe systemic diseases; (5) PHACE syndrome or major congenital anomalies.

### Randomization, allocation concealment, and blinding

Eligible participants were randomly assigned (1:1) to either the combination therapy group or the propranolol monotherapy group using a computer-generated random number sequence. Allocation concealment was ensured using sequentially numbered, sealed, opaque envelopes. Due to the nature of the interventions, participants and treating physicians were not blinded. However, outcome assessors and data analysts were blinded to the treatment assignments.

### Interventions

Monotherapy Group (n = 130): Participants received oral propranolol hydrochloride solution at a dose of 1 mg/kg/day, administered in two divided doses for 6 months.

Combination Group (n = 130): Participants received the same oral propranolol regimen plus monthly intralesional injections of bleomycin (1 mg/mL solution) for up to 6 months. The total dose did not exceed 1 mg/kg per session. The dose per injection site was standardized based on tumor volume: lesions <2 cm^3^ received 0.2 mg; lesions 2–5 cm^3^ received 0.3–0.4 mg; and lesions >5 cm^3^ received 0.5 mg, with multiple sites injected as needed. Injections were performed under local anesthesia using a topical lidocaine-prilocaine cream by an experienced dermatologist.

All participants underwent baseline cardiac evaluation (ECG, echocardiogram) and regular monitoring.

### Outcomes and assessments

Follow-up was conducted at baseline, monthly for 6 months, and at the end of treatment. The primary outcome was the excellent clinical therapeutic effect at 6 months.

Primary Endpoint: Tumor dimensions were measured by ultrasound to calculate volume (Volume = length × width × height × 0.52). Therapeutic effect was categorized as: Complete Regression (>95% volume reduction), Marked Effectiveness (≥75%–95%), Moderate Effectiveness (30% to <75%), or Ineffective (<30%). The primary endpoint was the combined rate of “Complete Regression” and “Marked Effectiveness”.

Secondary Endpoints: 1) Early Tumor Surface Response (at 24 h), graded as Obvious Atrophy, Mild Atrophy, or No Obvious Change. 2) Hemangioma Color Evaluation using a 4-point Likert scale (0–3). 3) Scar Assessment using the Vancouver Scar Scale (VSS) at 6 months. We chose the VSS over other scales like the Hemangioma Severity Scale (HSS) because our focus was specifically on the long-term cosmetic outcome and residual skin quality after treatment, for which the VSS is the gold standard. 4) Adverse Events (AEs), graded via CTCAE v5.0.

### Statistical analysis

Sample size was estimated based on an expected excellent response rate of 75% in the combination group and 55% in the monotherapy group (α = 0.05, power = 80%), requiring 127 participants per group. We enrolled 130 per group to account for dropouts. Data were analyzed using R software (version 4.2.1). Continuous variables were compared using t-tests or Mann-Whitney U tests. Categorical variables were compared using Chi-square tests, or Fisher’s exact test when expected cell counts were less than 5, such as for the adverse event analysis. Relative risk (RR) and 95% confidence intervals (CI) were calculated. P < 0.05 was considered significant. Analysis was on an intention-to-treat (ITT) basis.

## Results

### Participant disposition and baseline characteristics

**FIGURE 1:**
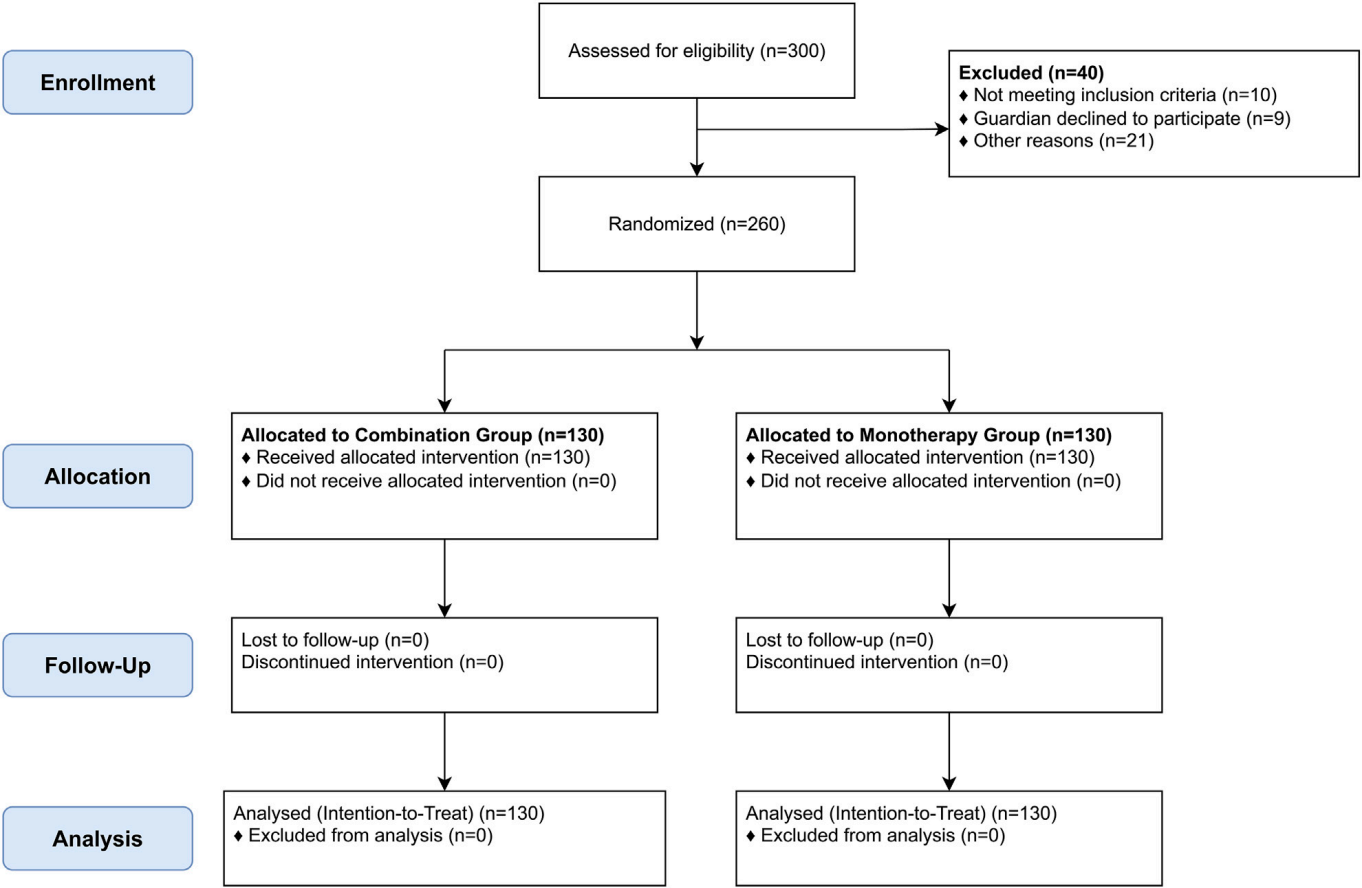
CONSORT 2010 flow diagram of participant enrollment, allocation, follow-up, and analysis.

From January 2024 to January 2025, 300 infants were assessed for eligibility. Forty were excluded (details in Table 1), and 260 were randomized (130 per group). All 260 randomized participants completed the 6-month follow-up and were included in the analysis (Figure 1). Baseline demographic and clinical characteristics were well-balanced between the two groups (Table 2).

**TABLE 1:** Reasons for exclusion of participants assessed for eligibility (n = 40).

Reason for exclusion	Number of patients (n)	Percentage (%)
Did not meet inclusion criteria	10	25.0%
- Age outside of 3–11 months range	6	
- Prior treatment received	4	
Guardian declined to participate	9	22.5%
Other reasons	21	52.5%
- Presence of significant comorbidity (e.g., cardiac)	8	
- Logistical issues (e.g., family lived too far away)	7	
- Failed cardiac screening	6	
Total Excluded	40	100%

**TABLE 2:** Baseline demographic and clinical characteristics of participants.

Characteristic	Monotherapy group (n = 130)	Combination group (n = 130)	Test statistic (t/χ^2^)	P-value
Age (months), mean ± SD	5.16 ± 2.33	5.49 ± 3.07	1.005 (t)	0.316
Sex (Male: Female), n	62:68	60:70	0.097 (χ^2^)	0.755
Tumor site, n (%)				
Head and Neck	78 (60.00%)	82 (63.07%)	0.269 (χ^2^)	0.604
Trunk	32 (24.61%)	35 (26.92%)	0.234 (χ^2^)	0.629
Limbs	20 (15.39%)	13 (10.00%)	2.273 (χ^2^)	0.132

### Primary endpoint: clinical therapeutic efficacy at 6 months

**FIGURE 2:**
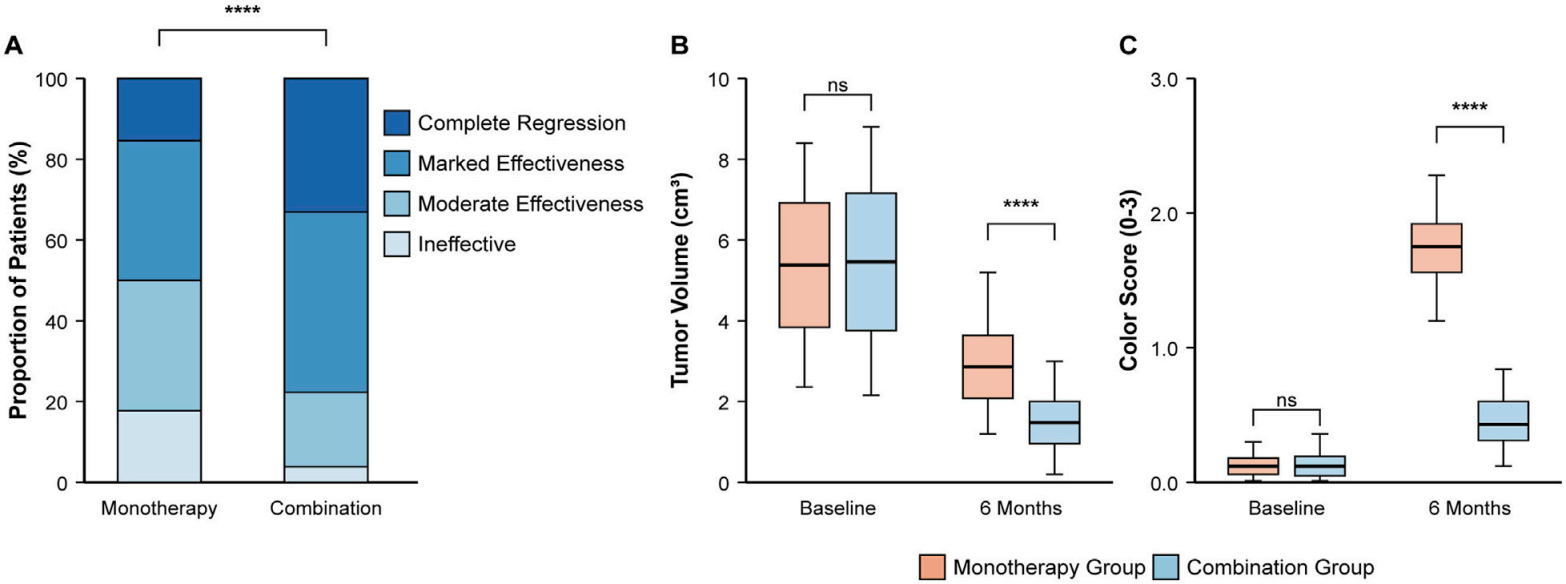
Comparison of primary and key secondary efficacy outcomes at 6 months. **(A)** Distribution of clinical therapeutic effects, showing a higher proportion of excellent responses in the combination group. **(B)** Boxplot showing tumor volume (cm^3^) at baseline and 6 months. **(C)** Boxplot showing hemangioma color scores at baseline and 6 months. Boxplots display the median, interquartile range, and whiskers. ****P < 0.0001 based on Chi-square test for **(A)** and independent t-test for **(B,C)** comparisons at 6 months.

The combination therapy demonstrated markedly superior efficacy after 6 months of treatment (Table 3; Figure 2A). The proportion of patients achieving an excellent therapeutic response was significantly higher in the combination group than in the monotherapy group (77.69% vs. 50.00%; RR, 1.55; 95% CI, 1.25 to 1.93; P < 0.001). This was driven by a significantly higher rate of complete regression in the combination group (33.07% vs. 15.38%; P = 0.001). Consequently, treatment was deemed ineffective in a significantly smaller percentage of patients in the combination group (3.85% vs. 17.69%; P < 0.001).

**TABLE 3:** Comparison of clinical therapeutic effects at 6 Months.

Outcome category	Monotherapy group (n = 130)	Combination group (n = 130)	χ^2^ value	P-value
Complete Regression, n (%)	20 (15.38%)	43 (33.07%)	10.783	0.001
Marked Effectiveness, n (%)	45 (34.61%)	58 (44.61%)	2.997	0.083
Moderate Effectiveness, n (%)	42 (32.31%)	24 (18.46%)	6.326	0.012
Ineffective, n (%)	23 (17.69%)	5 (3.85%)	13.031	<0.001
Excellent Response (Complete + Marked), n (%)	65 (50.00%)	101 (77.69%)	21.849	<0.001

### Secondary endpoints

#### Early tumor surface response within 24 hours

An early therapeutic response was observed in both groups, but the effect was more rapid and pronounced in the combination group (Table 4). While a majority of infants in both groups showed some improvement, and the overall proportion showing any response (obvious or mild atrophy) was not statistically different (79.23% in the combination group vs. 74.61% in the monotherapy group; P = 0.377), the key difference was in the degree of response. A significantly greater proportion of patients in the combination group exhibited “Obvious Atrophy” (40.00% vs. 14.61%; P < 0.001), indicating a more potent initial effect of the combination therapy.

**TABLE 4:** Early tumor surface response within 24 hours after first treatment.

Response grade	Monotherapy group (n = 130)	Combination group (n = 130)	Overall χ^2^ value	Overall P-value
Obvious Atrophy, n (%)	19 (14.61%)	52 (40.00%)	23.951	<0.001
Mild Atrophy, n (%)	78 (60.00%)	51 (39.23%)		
No Obvious Change, n (%)	33 (25.39%)	27 (20.77%)		
Any Response (Obvious + Mild), n (%)	97 (74.61%)	103 (79.23%)	0.781	0.377

The overall χ^2^ value and P-value correspond to the comparison of the distribution across the three response grades between the groups. While the overall proportion of patients showing “Any Response” was not statistically different, the combination group showed a significantly greater degree of response (i.e., more patients with “Obvious Atrophy”).

#### Tumor volume and color score reduction

After 6 months, the combination group showed significantly greater improvement in both tumor volume and color (Table 5). The mean tumor volume was approximately half that of the monotherapy group (1.63 ± 0.70 cm^3^ vs. 3.27 ± 1.06 cm^3^; P < 0.001) (Figure 2B). Similarly, the hemangioma color score improved more significantly, indicating more complete color fading (mean score at 6 months, 0.43 ± 0.29 vs. 1.25 ± 0.41; P < 0.001) (Figure 2C).

**TABLE 5:** Hemangioma color score.

Time point	Monotherapy group (n = 130)	Combination group (n = 130)	t-value	P-value
Baseline, mean ± SD	2.50 ± 0.35	2.53 ± 0.44	−0.648	0.518
After 6 Months, mean ± SD	1.25 ± 0.41	0.43 ± 0.29	18.412	<0.001

#### Residual scarring assessment

**FIGURE 3:**
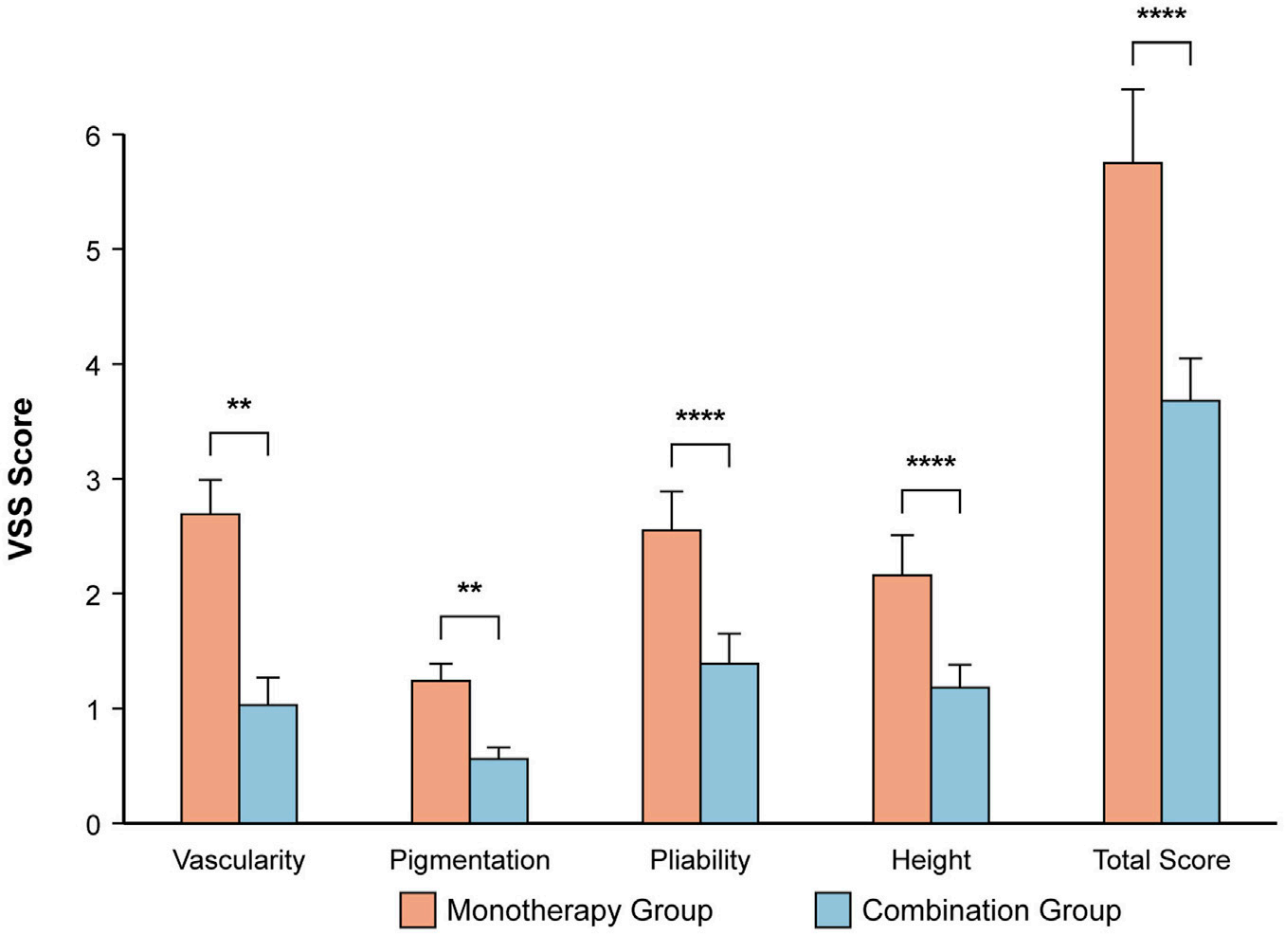
Assessment of residual scarring using the Vancouver Scar Scale (VSS) at 6 months. The bar chart displays the mean ± SD for each VSS component and the total score. ****P < 0.0001, **P < 0.01 based on independent t-test. The combination group showed significantly lower scores, indicating a better cosmetic outcome.

At the 6-month follow-up, the combination group had a superior cosmetic outcome with less residual scarring (Table 6; Figure 3). The mean total VSS score was significantly lower compared to the monotherapy group (3.68 ± 0.37 vs. 5.75 ± 0.64; P < 0.001). Significant improvements were seen in all VSS subscales (all P < 0.001).

**TABLE 6:** Vancouver scar scale (VSS) scores at 6 months.

VSS component	Monotherapy group (n = 130)	Combination group (n = 130)	t-value	P-value
Vascularity, mean ± SD	1.69 ± 0.30	1.03 ± 0.24	19.108	<0.001
Pigmentation, mean ± SD	1.24 ± 0.15	0.56 ± 0.10	43.167	<0.001
Pliability, mean ± SD	2.55 ± 0.34	1.39 ± 0.26	30.820	<0.001
Height, mean ± SD	2.16 ± 0.35	1.18 ± 0.20	26.680	<0.001
Total VSS Score, mean ± SD	5.75 ± 0.64	3.68 ± 0.37	31.841	<0.001

#### Safety and adverse events

The overall incidence of AEs was low and comparable between groups (Table 7). Local, transient redness and pain at the injection site were reported in 11/130 participants (8.46%) in the combination group. Systemic AEs possibly related to propranolol were infrequent and occurred at similar rates. No serious AEs (Grade ≥3) necessitating treatment discontinuation were reported.

**TABLE 7:** Incidence of adverse events during treatment.

Adverse event	Monotherapy group (n = 130)	Combination group (n = 130)	χ^2^ value	P-value
Local Redness and Pain, n (%)	0 (0%)	11 (8.46%)	11.450	<0.001
Bradycardia (transient), n (%)	3 (2.31%)	4 (3.08%)	0.149	0.700
Hypotension (mild, transient), n (%)	0 (0%)	1 (0.77%)	1.004	0.316
Hypoglycemia (mild, transient), n (%)	0 (0%)	2 (1.54%)	2.016	0.156

## Discussion

This RCT demonstrates that augmenting a low-dose oral propranolol regimen (1 mg/kg/day) with monthly intralesional bleomycin is significantly more effective than low-dose monotherapy for treating IH in infants. The combination therapy resulted in a higher rate of excellent clinical response, faster tumor atrophy, greater improvement in color and volume, and less residual scarring, all with a comparable safety profile.

The enhanced efficacy likely stems from synergistic actions. Propranolol exerts systemic effects, while intralesional bleomycin acts locally as a sclerosing agent (Muir et al., 2004; Kumar et al., 2021). Our finding of a more pronounced tumor response within 24 h supports the rapid, potent local action of bleomycin. Beyond their independent actions, a deeper synergy may exist at the cellular level. Propranolol’s inhibition of VEGF signaling not only curtails angiogenesis but may also normalize the tumor microenvironment, potentially making the aberrant endothelial cells more susceptible to bleomycin-induced DNA damage and apoptosis (Mungunsukh et al., 2010). This concept of cellular sensitization, where β-blockers enhance chemotherapy efficacy, has been explored in other oncologic contexts and may explain the accelerated regression we observed (Filippi et al., 2020; Puzderova et al., 2023).

An intriguing aspect of our findings is the superior scar outcome in the combination group, despite bleomycin’s fibrosing mechanism. We postulate that this is due to the nature of the induced fibrosis. The rapid and controlled involution promoted by the combination therapy may lead to a more organized, structured fibrosis and tissue remodeling, contrasting with the protracted inflammation and disorganized healing that can occur in large, slowly involuting hemangiomas treated with monotherapy (Luo, 2023). Thus, the therapy may replace a pathologic process with a more controlled wound-healing response, ultimately resulting in a better cosmetic appearance (Luo, 2023).

Our study has several strengths, including its randomized design, blinded outcome assessment, and use of an ITT analysis. However, limitations must be acknowledged. First, while we have now clarified the bleomycin dosing protocol, the dose was still titrated based on baseline volume rather than a fixed dose for all, which could introduce subtle variability. Second, we acknowledge the exceptional 100% follow-up rate and have addressed the likely contributing factors, though this may limit generalizability to settings with less intensive follow-up. The exceptional follow-up rate is attributed to a combination of factors including a highly motivated patient cohort, intensive follow-up coordination by a dedicated study nurse, and the provision of all treatments free of charge. Third, to minimize measurement variability in ultrasound, all assessments were performed by one of two senior radiologists, and a *post hoc* analysis revealed high inter-rater reliability [Intraclass Correlation Coefficient (ICC) = 0.96]. Finally, as a single-center study with a 6-month follow-up, generalizability is limited, and longer-term outcomes are unknown.

## Conclusion

In conclusion, for infants with IH, augmenting a low-dose oral propranolol regimen (1 mg/kg/day) with monthly intralesional bleomycin is significantly more effective than low-dose propranolol monotherapy. This combination strategy accelerates tumor regression and yields superior cosmetic outcomes by reducing scarring, all while maintaining a comparable safety profile. This approach presents a valuable clinical option for optimizing treatment, particularly when seeking to enhance efficacy while utilizing a lower systemic dose of propranolol.

## Data Availability

The original contributions presented in the study are included in the article/supplementary material, further inquiries can be directed to the corresponding author.

## Abbreviations

AE, Adverse Event; CI, Confidence Interval; CONSORT, Consolidated Standards of Reporting Trials; ECG, Electrocardiogram; IH, Infantile Hemangioma; ITT, Intention-To-Treat; RCT, Randomized Controlled Trial; RR, Relative Risk; SD, Standard Deviation; VEGF, Vascular Endothelial Growth Factor; VSS, Vancouver Scar Scale.

## References

[B1] Chen T. Gudipudi R. Nguyen S. A. Carroll W. Clemmens C. (2023). Should propranolol remain the Gold standard for treatment of infantile hemangioma? A systematic review and meta-analysis of propranolol Versus atenolol. Ann. Otology, Rhinology, Laryngology 132 (3), 332–340. 10.1177/00034894221089758 35466712

[B2] Chen Q. Rong H. Zhang L. Wang Y. Bian Q. Zheng J. (2024). KLF2 orchestrates pathological progression of infantile Hemangioma through Hemangioma stem cell fate decisions. J. Investigative Dermatology 144 (8), 1850–64.e9. 10.1016/j.jid.2024.01.029 38382868

[B3] Filippi L. Bruno G. Domazetovic V. Favre C. Calvani M. (2020). Current therapies and new targets to fight melanoma: a promising role for the β3-Adrenoreceptor. Cancers 12 (6), 1415. 10.3390/cancers12061415 32486190 PMC7352170

[B4] Guo L. Wang M. Song D. Sun J. Wang C. Li X. (2024). Additive value of single intralesional bleomycin injection to propranolol in the management of proliferative infantile hemangioma. Asian J. Surg. 47 (1), 154–157. 10.1016/j.asjsur.2023.05.170 37328380

[B5] Hermans M. M. Schappin R. de Laat P. C. J. Mendels E. J. Breur J. Langeveld H. R. (2024). Mental health of school-aged children treated with propranolol or atenolol for infantile hemangioma and their parents. Dermatology 240 (2), 216–225. 10.1159/000536144 38228125 PMC10997238

[B6] Hoeger P. H. Harper J. I. Baselga E. Bonnet D. Boon L. M. Ciofi Degli Atti M. (2015). Treatment of infantile haemangiomas: recommendations of a European expert group. Eur. J. Pediatr. 174 (7), 855–865. 10.1007/s00431-015-2570-0 26021855

[B7] Horbach S. E. R. Rigter I. M. Smitt J. H. S. Reekers J. A. Spuls P. I. van der Horst C. (2016). Intralesional bleomycin injections for vascular malformations: a systematic review and meta-analysis. Plastic Reconstr. Surg. 137 (1), 244–256. 10.1097/PRS.0000000000001924 26710030

[B8] Kiani I. Khalili M. Abdollahimajd F. Eshghi P. Khameneh Bagheri A. (2025). Ultrasound-guided percutaneous sclerotherapy with bleomycin for management of infantile subcutaneous hemangioma: a case report. Clin. Case Rep. 13 (2), e70143. 10.1002/ccr3.70143 39944862 PMC11813699

[B9] Krowchuk D. P. Frieden I. J. Mancini A. J. Darrow D. H. Blei F. Greene A. K. (2019). Clinical practice guideline for the management of infantile hemangiomas. Pediatrics 143 (1), e20183475. 10.1542/peds.2018-3475 30584062

[B10] Kumar R. Tiwari P. Pandey V. Kar A. G. Tiwary N. Sharma S. P. (2021). A clinicopathological study to assess the role of intralesional sclerotherapy following propranolol treatment in infantile hemangioma. J. Cutan. Aesthetic Surg. 14 (4), 409–415. 10.4103/JCAS.JCAS_103_20 35283595 PMC8906266

[B11] Léauté-Labrèze C. Harper J. I. Hoeger P. H. (2017). Infantile haemangioma. Lancet (London, England) 390 (10089), 85–94. 10.1016/s0140-6736(16)00645-0 28089471

[B12] Lee D. Boscolo E. Durham J. T. Mulliken J. B. Herman I. M. Bischoff J. (2014). Propranolol targets the contractility of infantile haemangioma-derived pericytes. Br. J. Dermatology 171 (5), 1129–1137. 10.1111/bjd.13048 24720697 PMC4193942

[B13] Lu W. Yang Z. Wang M. Zhang Y. Qi Z. Yang X. (2024). Identification of potential therapeutics for infantile hemangioma via *in silico* investigation and *in vitro* validation. Drug Des. Dev. Ther. 18, 4065–4088. 10.2147/DDDT.S460575 39286286 PMC11404501

[B14] Luo Q. F. (2023). The combined application of bleomycin and triamcinolone for the treatment of keloids and hypertrophic scars: an effective therapy for treating refractory keloids and hypertrophic scars. Skin. Res. Technol. 29 (6), e13389. 10.1111/srt.13389 37357650 PMC10244805

[B15] Muir T. Kirsten M. Fourie P. Dippenaar N. Ionescu G. O. (2004). Intralesional bleomycin injection (IBI) treatment for haemangiomas and congenital vascular malformations. Pediatr. Surg. Int. 19 (12), 766–773. 10.1007/s00383-003-1058-6 14740248

[B16] Mungunsukh O. Griffin A. J. Lee Y. H. Day R. M. (2010). Bleomycin induces the extrinsic apoptotic pathway in pulmonary endothelial cells. Am. J. Physiol. Lung Cell Mol. Physiol. 298 (5), L696–L703. 10.1152/ajplung.00322.2009 20154224 PMC2867402

[B17] Puzderova B. Belvoncikova P. Grossmannova K. Csaderova L. Labudova M. Fecikova S. (2023). Propranolol, promising chemosensitizer and candidate for the combined therapy through disruption of tumor microenvironment homeostasis by decreasing the level of carbonic anhydrase IX. Int. J. Mol. Sci. 24 (13), 11094. 10.3390/ijms241311094 37446271 PMC10341831

[B18] Schulz K. F. Altman D. G. Moher D. CONSORT Group (2010). CONSORT 2010 statement: updated guidelines for reporting parallel group randomised trials. BMJ Clin. Res. Ed. 340, c332. 10.1136/bmj.c332 20332509 PMC2844940

[B19] Sharma A. Gupta M. Mahajan R. (2024). Infantile hemangiomas: a dermatologist’s perspective. Eur. J. Pediatr. 183 (10), 4159–4168. 10.1007/s00431-024-05655-8 39052139

[B20] Storch C. H. Hoeger P. H. (2010). Propranolol for infantile haemangiomas: insights into the molecular mechanisms of action. Br. J. Dermatology 163 (2), 269–274. 10.1111/j.1365-2133.2010.09848.x 20456345

[B21] Tiwari P. Pandey V. Bera R. N. Tiwary N. Mishra A. Sharma S. P. (2022). Sandwich therapy in the management of propranolol resistant infantile hemangioma of the lip. J. Stomatology, Oral Maxillofac. Surg. 123 (5), e499–e505. 10.1016/j.jormas.2022.02.010 35217221

[B22] Zavras N. Dimopoulou A. Machairas N. Paspala A. Vaos G. (2020). Infantile hepatic hemangioma: current state of the art, controversies, and perspectives. Eur. J. Pediatr. 179 (1), 1–8. 10.1007/s00431-019-03504-7 31758313

